# In-Vitro Screenings for Biological and Antioxidant Activities of Water Extract from *Theobroma cacao* L. Pod Husk: Potential Utilization in Foods

**DOI:** 10.3390/molecules26226915

**Published:** 2021-11-16

**Authors:** Mustanir Yahya, Binawati Ginting, Nurdin Saidi

**Affiliations:** Department of Chemistry, Faculty of Mathematics and Natural Sciences, Universitas Syiah Kuala, Banda Aceh 23111, Indonesia; binawati@unsyiah.ac.id (B.G.); nurdin@unsyiah.ac.id (N.S.)

**Keywords:** *Theobroma cacao*, aqueous extract, pod husk, cocoa, antibacterial, antifungal, cytotoxicity, antioxidant, phytosterol

## Abstract

Increasing production of cocoa (*Theobroma cacao* L.) leads to a higher environmental burden due to its solid waste generation. Cocoa pod husk, one of the major solid wastes of cocoa production, contains rich bioactive compounds unveiling its valorization potential. With that in mind, our research aimed to explore the biological and antioxidant activities of aqueous extracts from cocoa pod husks. In this present work, cocoa pod husk was extracted using water and subsequentially partitioned using n-hexane, ethyl acetate, and methanol. The antimicrobial investigation revealed that the ethyl acetate solubles were active against the *Staphylococcus aureus*, *Escherichia coli*, and *Candida albicans*, where at a 20% *w*/*v* concentration, the inhibition diameters were 6.62 ± 0.10, 6.52 ± 0.02, and 11.72 ± 0.36 mm, respectively. The extracts were found non-toxic proven by brine shrimp lethality tests against *Artemia salina* with LC_50_ scores ranging from 74.1 to 19,054.6 μg/mL. The total phenolic content and total flavonoid content were obtained in the range of 47.44 to 570.44 mg/g GAE and 1.96 to 4.34 mg/g QE, respectively. Antioxidant activities of the obtained extracts were revealed by 2,2-diphenyl-1-picryl-hydrazyl-hydrate (DPPH) assay with EC_50_ reached as low as 9.61 μg/mL by the ethyl acetate soluble. Phytochemical screening based on gas chromatography—mass spectroscopy analysis on the sample with the highest antioxidant activities revealed the dominant presence of three phytosterols, namely gamma-sitosterol, stigmasterol, and campesterol.

## 1. Introduction

Cocoa (*Theobroma cacao* L.) is recognized as a vital export commodity for some countries, such as Ghana, Cote D’Ivoire, Indonesia, Cameroon, and Nigeria. Its production is maintained in increasing trend, with its compound annual growth rate (CAGR) reaching 7.3% from 2019 to 2025 [1]. The production itself, in 2019/2020, was reported to be 4697 tons by the International Cocoa Organization [2]. Each ton of dried cocoa beans production was estimated to produce 10 tons of cocoa pod husk, threatening to add environmental burdens [3]. To support the sustainability of the agricultural sector, many efforts have been conducted to valorize cocoa by-products.

The recovery of bioactive compounds from cocoa pod husk has been considered as a strategic valorization approach [4]. On one hand, secondary metabolites from *T. cacao* are shown to possess high antioxidant activities [5,6]. This is ascribed to the high content of polyphenols reported by various works [7]. Additionally, antimicrobial activities of *T. cacao* against various pathogens (such as *Staphylococcus aureus* and *Escherichia coli*) have been reported as well [8]. On the other hand, cocoa pod husk also exhibits high antioxidant activities and biological activities due to a similar phytoconstituents profile with that of cocoa bean [9,10,11]. Moreover, the valorization of cocoa pod husk in the diet has been well-elaborated in a published review [12]. Hence, cocoa pod husk can act as the source of bioactive and antioxidant compounds which hold significance in foods [13,14].

Previously, researchers had investigated cocoa pod husk using various solvents, such as methanol:acetone [9], ethyl acetate [10], and n-hexane solvents [11]. Its extraction had also been conducted with the Soxhlet technique [15] and microwave-assisted techniques [16]. Despite many published studies reporting on cocoa pod husk, its aqueous extract along with partition method is scarcely investigated. Herein, the cocoa pod husk was extracted using water and followed by partition using solvents with increasing polarity. The methods employed in this work allow a thorough screening of antioxidant and biological activities of the extract, followed by phytoconstituent identification.

## 2. Materials and Methods

### 2.1. Chemicals

Chemicals used in this study included concentrated ammonia, methanol, ethyl acetate, n-hexane, gelatin, FeCl_3_, NH_3_, chloroform, concentrated HCl, HCl 0.5 M, Mg metal, Mueller Hinton Agar (MHA), Nutrient Agar (NA), Sabouraud Dextrose Agar (SDA), gentamicin, ketoconazole, ethanol 70 and 96%, 2,2-diphenyl-1-picrylhydrazyl (DPPH), ascorbic acid, NaHCO_3_, gallic acid, AlCl_3_, and quercetin. For qualitative screening of the phytochemical contents, the following reagents were used: Liebermann–Burchard, Dragendorff, Mayer and Wagner’s reagents. In the determination of total flavonoids content, Folin–Ciocalteu reagent was used. All of the chemicals were procured from Merck (Selangor, Malaysia) and analytical grade.

### 2.2. Plant Specimen

Plant specimens of *Theobroma cacao* L. and its pod husks were collected from Kutacane, Aceh, Indonesia in March 2020 and identified in the Department of Biology, Universitas Syiah Kuala, Indonesia (No. 795/UN11.1.28.1/DT/2008). The pod husks were sequentially washed using distilled water, cut into small pieces, and air-dried at room temperature (25 ± 1 °C) for 7 to 10 days. The sample was then crushed to obtain its powder for maceration.

### 2.3. Extraction of T. cacao Pod Husks

**Scheme 1:**
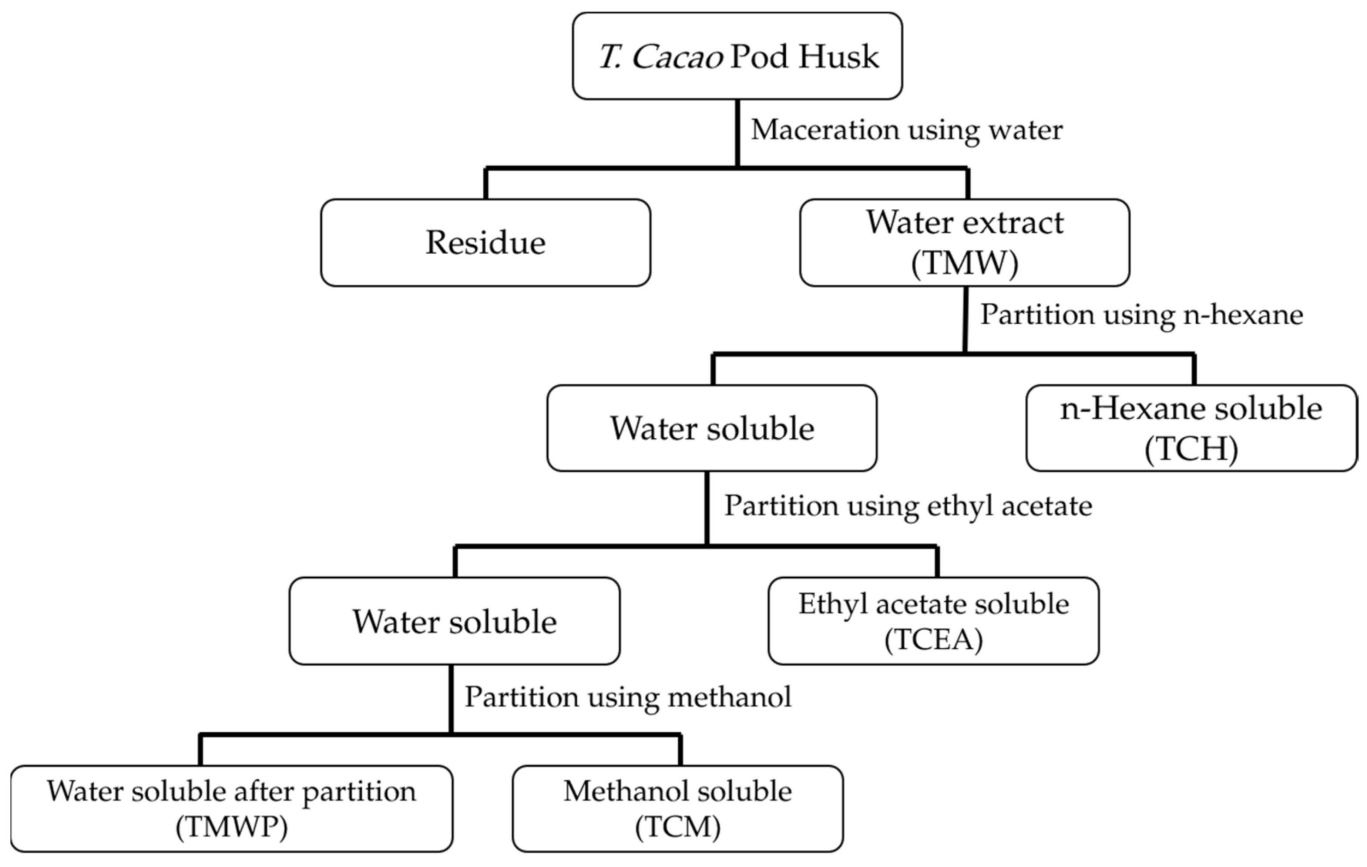
*T. cacao* pod husk extraction using water and partition using n-hexane, ethyl acetate, and methanol.

Cocoa pod husk powder (1.5 kg) was macerated at room temperature using distilled water for 1 to 3 h repeatedly so a clear filtrate was obtained. The filtrate was concentrated using a rotary evaporator to produce concentrated water extract from T. cacao pod husk (TCW). Thereafter, the extract was sequentially partitioned using n-hexane, ethyl acetate, and methanol to yield n-hexane (TCH), ethyl acetate (TCEA), and methanol solubles (TCM), respectively. At the end of the partition, the remaining aqueous soluble was labeled as the water partition of T. cacao pod husk (TCWP) (Scheme 1). All samples, TCW, TCH, TCEA, TCM, and TCWP were screened qualitatively for their phytoconstituents, per the suggestion of previous literature [11].

### 2.4. Qualitative Phytochemical Analysis

The phytochemical analyses on the presence of alkaloids, terpenoids, steroids, saponins, flavonoids and phenolics in the samples were conducted qualitatively [17]. For alkaloids, 50 mg of sample was drop-wised with concentrated NH_3_ (2 mL) and chloroform (5 mL), subsequently filtered and concentrated. Into the filtrate, 5 mL HCl 5% was added, shaken and left aside until two layers were formed. The dissolved layer was separated into three test tubes. Into each test tube, Mayer, Wagner, and Dragendorff’s reagents were added, where positive results would be indicated by the formation of white, yellow, and brown-reddish precipitates, respectively.

As for terpenoids and steroids, 50 mg of sample was tested with Liebermann–Burchard reagent. The presence of terpenoids would be indicated by the color changes to purple or red. Meanwhile, if the color changed to green or blue, it indicated steroids. Saponins were identified by dissolving a 20 mg sample into pre-heated methanol, which was subsequently extracted using ethyl acetate. The undissolved fraction was then added to 5 mL of water and shaken intensely, the presence of foam lasting for ±30 min suggested saponin contents.

Flavonoids were detected in the sample (50 mg) through its reaction with Mg powder and concentrated HCl (0.5 mL) concomitant to its dissolution in 3 mL pre-heated methanol (50%). The color changing into red, or purple suggested that the sample contained flavonoids. As for phenolic contents, the analysis was conducted using a ferric chloride test, where a few drops (three to four drops) of FeCl_3_ solution (5% *w*/*v*) were drop-wised onto the sample. The formation of bluish or black color indicated the phenolic contents in the sample.

### 2.5. Disc Diffusion Assay

Antimicrobial activities of the obtained extracts were conducted based on the disc diffusion assay using gram-positive *Staphylococcus aureus*, gram-negative *Escherichia coli*, and *Candida albicans*. Prior to the test, all apparatus were sterilized using an autoclave (120–150 °C). As much as 30 mL MHA and SDA media (for antibacterial and antifungal tests, respectively) were prepared and solidified onto a petri dish. The microorganisms were suspended in 5 mL NaCl 0.9% and swabbed onto the prepared MHA media. The media was divided into six segments, where onto each segment a disc with diffused extract was placed. The concentration of the extract was ranged from 1 to 20% *w*/*v*. Antibiotic gentamicin and solvent (20 μL) were used as positive and negative controls, respectively. Afterward, the incubation was carried out at 37 °C for 24 h before the inhibition zone diameter was measured. The bacterial inhibition growth was expressed in inhibition diameter (mm).

### 2.6. Brine Shrimp Lethality Test (BSLT) Assay

Filtered seawater was prepared in a container separated into two parts and connected with one tunnel, where one part was made dark and the other was lit using 15–20 Watt lamp. Brine shrimp eggs (*Artemia salina*) were placed in the dark part of the container to hatch (25–29 °C; 48 h) and drawn to the lighted part once hatched. Extract samples (30 mg) were dissolved using a mixture of DMSO 5% (*w*/*v*) in 30 mL of filtered seawater and subsequently sonicated until homogenous. The extract concentration ranged from 500 to 1 μg/mL, where seawater containing only DMSO 5% (*w*/*v*) was used as a negative control. Each sample was placed into a glass container followed by the addition of 10 brine shrimp and incubated for 24 h at room temperature. Dead brine shrimp were indicated by the absence of movement and counted. The results were expressed in LC_50_—the minimum required concentration to obtain 50% lethality. This assay was conducted in triplicate.

### 2.7. Determination of Total Phenolic Content

Extracts were firstly dissolved in distilled water, pipetted as much as 0.2 mL, and added into a mixture of distilled water (15.8 mL) and Folin–Ciocalteu reagent (1 mL). After shaking, the solution was left for 8 min, and then added to Na_2_CO_3_ 10% and incubated for 2 h at room temperature. Each sample was measured for its absorbance using UV–Vis spectrophotometer (Shimadzu UVmini-1240, Kyoto, Japan) at λ = 765 nm. The measurement of total phenolic content (TPC) was carried out in triplicate, as suggested previously [18].

### 2.8. Determination of Total Flavonoid Content

Total flavonoid content (TFC) was determined in triplicate following the procedure reported previously [18]. The extract sample was dissolved in ethanol to obtain a solution with a concentration of 2, 4, 6, 8, and 10 μg/mL. Extract samples of each concentration were added to ethanol (3 mL), AlCl_3_ (0.2 mL), potassium acetate (0.2 mL), and distilled water (5.6 mL). Thereafter, the solution was incubated for 30 min at room temperature, before being measured for its absorbance using a UV-Vis spectrophotometer at a wavelength of 440 nm.

### 2.9. 2,2-Diphenyl-1-picrylhydrazyl Assay

Antioxidant activity of the extract was tested based on its scavenging ability against 2,2-diphenyl-1-picrylhydrazyl (DPPH) free radicals, where the procedure was conducted in triplicate. To start with, 7.9 mg DPPH powder with a molecular weight of 394.32 g/mol was dissolved in methanol with a total volume of 50 mL in a volumetric flask. The sample extracts were varied in concentration; 6.25, 12.5, 25, 50, and 100 μg/mL with a volume of 62.5, 125, 250, 500, and 1000 μL, respectively. Into each prepared extract, 1 mL DPPH 0.4 mM was added, and the volume was made up to 5 mL using methanol. Each solution was homogenized using a vortex mixer and incubated (30 min; 37 °C). All absorbances of the samples were recorded at 517 nm using a UV-Vis spectrophotometer. A similar procedure was repeated with ascorbic acid with a concentration ranging from 1 to 6 μg/mL. The results were presented in inhibition (%) and EC_50_—a minimum concentration required to yield 50% scavenging activity.

Extract samples yielding the highest antioxidant activities were analyzed for phytoconstituents using Gas Chromatography–Mass Spectrometry (GC-MS) (Shimadzu QP2000A, Kyoto, Japan).

### 2.10. Data Analysis

Statistical analyses on the obtained data were carried out on GraphPad Prism 9.2.0 software (GraphPad Software, San Diego, CA, USA). The data were firstly tested for their normal distribution using the Shapiro–Wilk test. Statistical significance was obtained through *t*-test or one-way analysis of variance (ANOVA).

## 3. Results and Discussion

### 3.1. Yield and Phytochemical Contents

Yields obtained from the extraction of cocoa pod husk using various solvents have been presented (Table 1). TCW had the highest yield percentage (33.402%) among other extracts showing the abundance of phytoconstituents in the extract. Furthermore, TCWP had the second-highest extract yield. Water extracts have been reported to draw a wide spectrum of bioactive compounds from the plant. TCW, TCEA, TCM, and TCWP were shown positive containing alkaloids, flavonoids, saponins, and phenolics. Saponins along with most alkaloids and phenolics are polar, so therefore, could be easily dissolved in polar extract solvents. Additionally, TCEA was also found to contain terpenoids, which was similar to TCH. Meanwhile, steroids were only observed in TCH. Non-polar n-hexane and semi-polar ethyl acetate were reported capable of extracting terpenoids and steroids. The composition of phytoconstituents in an extract determines its bioactivities which will be discussed in the following sections.

### 3.2. Antibacterial Activities

Plant extracts with antibacterial properties could be used as an additive in foods to delay spoilage. Herein, the antibacterial activities of aqueous extracts from cocoa pod husk had been investigated against gram-positive *S. aureus* and gram-negative *E. coli*, where the results were presented in Table 2. Both of the bacteria are pathogenic and their availability is commonly used to determine the sanitary quality of foods [19]. Despite the presence of antibacterial secondary metabolites (such as alkaloids [20,21], saponins [22], and phenols [23]), TCW was observed to possess no inhibition effect against bacterial growth; even when the concentration was raised to 20% *w*/*v*. The impurities and antagonistic combination of phytoconstituents might be responsible for its inactive antibacterial properties.

The antibacterial activities against *S. aureus* were identified in TCM, TCEA, and TCWP, where at the concentration of 20%, the inhibition diameters were 6.38 ± 0.08, 6.62 ± 0.10, and 6.55 ± 0.04 mm. Furthermore, TCEA showed its inhibition effect against the growth of *S. aureus* at a concentration of 10% (6.14 ± 0.04 mm), and also was the only one that was effective against *E. coli* (at 20%; 6.52 ± 0.02 mm). Meanwhile, other extract samples did not exhibit an inhibition against gram-negative *E. coli* that possesses a double cellular membrane. These results suggest a higher concentration of antibacterial compounds contained in TCEA. However, our findings are different from the previously published work, where they investigated methanolic extract from cocoa pod husk and found higher antibacterial activities against *S. aureus* and *E. coli* in the crude extract and methanol soluble than that of the ethyl acetate soluble [8].

### 3.3. Antifungal Activities

Foods are susceptible to fungal contamination, becoming the main concern in the industry for food spoilage prevention [24]. In this study, antifungal activities of the extract were studied using *Candida albicans*. Candida sp. bacteria are common microorganisms in food spoilage and opportunistic pathogens for humans [25]. The antifungal activities against *C. albicans*, in this study, were only shown by TCH and TCEA (Table 2). Low activities of several extract samples could be due to the ability of *C. albicans* in forming a biofilm that provides protection against antifungals [26]. Inhibition diameter reaching 11.72 ± 0.36 mm was obtained from 20% TCEA. Meanwhile, TCH was found active even at a concentration of 5% (6.70 ± 0.10 mm), but relatively lower than TCEA when the concentration was increased to 20% (9.68 ± 0.02 mm). These activities could be associated with the presence of certain phytoconstituents in either TCH or TCEA. Previously, extracts from cacao plants have been reported to possess potent antimicrobial activity against *C. albicans* [27] along with several other fungi (such as *Aspergillus niger*, *Aspergillus flavus*, and *Trichophyton rubrum*) [28]. 

### 3.4. Cytotoxicity

The cytotoxicity test of the aqueous cocoa pod husk extracts was performed on *A. salina* with results presented in Table 3. The test revealed TCM had the lowest LC_50_ (74.1 μg/mL) suggesting the sample is the most cytotoxic among others. Following that, the cytotoxicity level from the highest to the lowest was shown by TCEA (104.7 μg/mL), TCW (5495.4 μg/mL), and TCH (19,054.6 μg/mL). Nonetheless, those values describe the extract samples as non-toxic since all of them are above 1 μg/mL [29,30]. Therefore, in terms of toxicity, extracts obtained in this research are eligible for food ingredients. Yet, as a disclaimer, results from BSLT should only be treated as an initial screening, where further investigation should be carried out to confirm the non-toxicity of the samples. For example, an assay using *Arthemia* spp. failed to indicate the toxicity of several extracted compounds against other species (*Chromis viridis*) [31].

In previous studies, similar ignorable cytotoxicity was obtained from the cacao plant extracts. For instance, the most cytotoxic fraction of ethyl acetate extract of cocoa pod husk only reached 107.15 μg/mL [10]. Lower cytotoxicity against *A. salina* was even reported for extracts obtained from cocoa pod husk with 70% ethanol (LC_50_ = 39,595.27 μg/mL) [32]. However, n-hexane extract of cocoa pod husk yielded the value of LC_50_ below 1 μg/mL (0.29 μg/mL) [11]. Collaboratively, current findings on the cytotoxicity of cacao plant extracts appeared differently depending on the solvent used for the extraction.

### 3.5. Total Phenolic Content (TPC)

**Figure 1:**
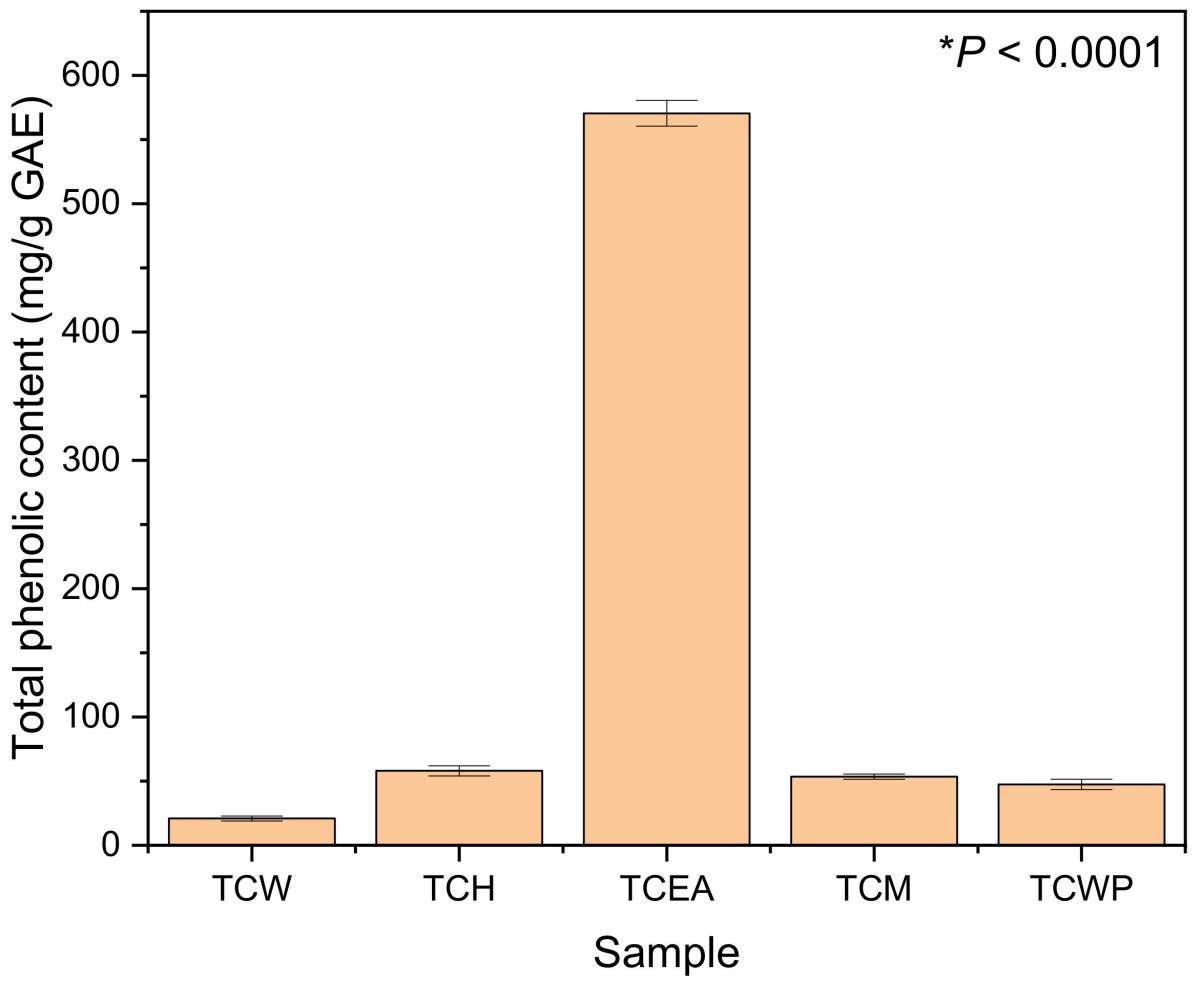
Total phenolic content of cocoa pod husk extracts. (*) statistically significant at *p* < 0.05 based on one-way ANOVA.

*T. cacao* has been recognized to contain rich phenolic phytoconstituents which could be utilized to prevent cancer through diets [33]. A clear depiction of the dominant TPC among other samples in this research was shown by TCEA (570.44 mg/g GAE) in Figure 1. In hierarchical order, after the TCEA, TCH (58 mg/g GAE) came second, followed by TCM (53.44 mg/g GAE), TCWP (47.44 mg/g GAE), and TCW (20.88 mg/g GAE). Extracts obtained directly from maceration using aqueous solvent had the lowest TPC because the content was dominated by inactive and inert compounds. However, this value is only slightly lower in comparison to that of cocoa pod husk extract using supercritical CO_2_ (23.2 ± 1.2 mg/g GAE) [15]. Moreover, a previous study reported lower TPC of aqueous extract from cocoa pods with heat-assisted following computational optimization [34]. These differences between reported studies might stem from the origin of the plant, where for the record, we collected the sample from the highland region—Kutacane, Aceh, Indonesia.

The highest TPC value in our present work was found to be relatively superior to a reported study which also valorized cacao by-products using either ethanol or methanol:acetone solvents [9]. Another study using Soxhlet extraction with n-hexane and ethanol on T. *cacao* bean hull also yielded a lower TPC value (58 ± 1 mg/g GAE) [15]. The TPC of our sample (TCEA) was even higher in comparison to the extracts from well-known antioxidant sources, such as mint, polyfloral, raspberry, sunflower, rape, and thyme [18]. TPC is an important parameter to predict antioxidant activity [35,36,37]. A study even found a significant correlation between the TPC and antibacterial activities [38].

### 3.6. Total Flavonoid Content (TFC)

**Figure 2:**
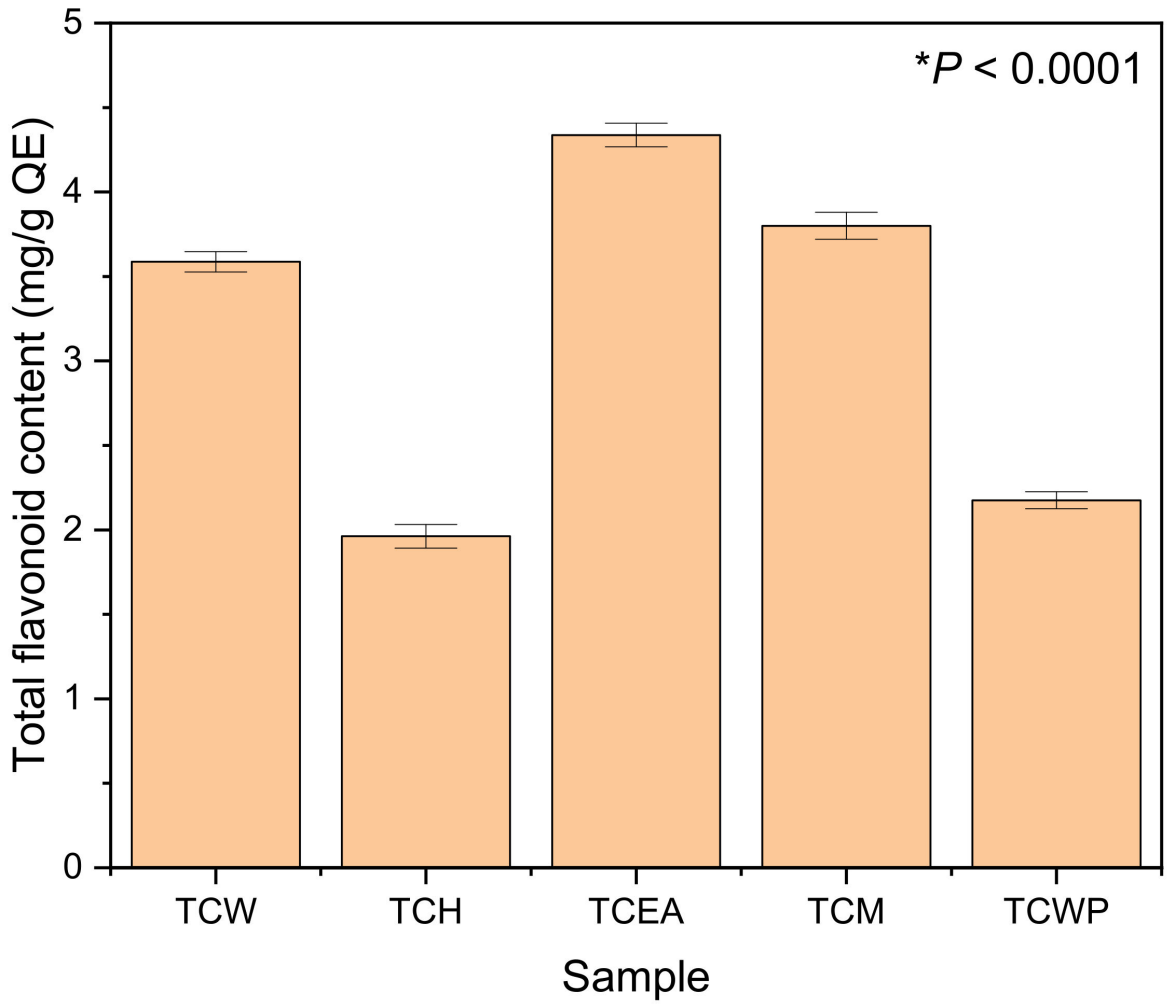
Total flavonoid content of cocoa pod husk extracts. (*) statistically significant at *p* < 0.05 based on one-way ANOVA.

TFC in this study was expressed in Quercetin equivalent (QE), where the values were found different in each sample (Figure 2). TFC in cocoa pod husk is scarcely reported, where related studies did not conduct the determination [9,15,34]. In increasing order, the TFC values of TCH, TCWP, TCW, TCM, and TCEA are 1.96, 2.18, 3.59, 3.80, and 4.34 mg/g QE, respectively. TFC in TCEA was found to be the highest which is in accordance with the fact that the sample contains the highest TPC. It is believed polyphenols, including flavonoids, have a strong affinity for semipolar solvents, including ethyl acetate [36,39]. However, its difference to TFC from other samples is not as significant as in TPC. We speculate that the phenolic compounds detected earlier were not from flavonoids.

These numbers are relatively low in comparison to extracts from other plants. For instance, methanol extracts from the popular ethnomedicinal plant *Senna singueana* could contain TFC in as much as 7.37 mg/g QE [40]. Water extract from *Eucalyptus camaldulensis* leaves collected from various locations in Burkina Faso had TFC ranging from 8.2 to 11.4 mg/g QE [37]. Higher TFC was even obtained by a published report extracting onion skin with methanol assisted with sonication assistance (up to 168.77 ± 0.87 mg/g QE) [35]. However, TFCs of our samples are still higher than that obtained from *Melia azedarach* (0.53 mg/g QE) and *Lannea discolor* leaves (0.15 mg/g QE) extraction using methanol [40]. TFCs could be different in extract samples depending on the solvent, part of the plant, and extraction technique. Antioxidant flavonoids are reported to be mostly produced in mesophyll cells in chloroplasts acting against endogenous ROS [41]. 

### 3.7. Antioxidant Activities

Antioxidant activities of aqueous extracts from the cocoa pod husk were investigated based on their DPPH scavenging activities (Table 4). The profile of EC_50_s of the sample in this analysis was similar to TPC values, where TCEA had the lowest EC_50_ and produced a wide gap with that of other samples. In line with a previously reported study, TPC is more definitive in predicting DPPH scavenging activity than TFC, evidenced by a higher correlation value [35,36,37]. Interestingly, TCW is the second most active among all samples, suggesting its potential as an alternative antioxidant source in food. TCM, TCWP, and TCH had EC_50_s of over 100 μg/mL (108.33, 115.52, and 116.70, respectively).

For comparison, the antioxidant activities of TCEA in this present work were found to be higher than in reports specializing in the antioxidant analysis of plant extract, including that of methanolic onion skin extracts [35]. TCEA was also more potent than that of the aqueous extract from *E. camaldulensis* in terms of scavenging DPPH free radicals [37]. Similarly, the antioxidant properties of well-known herbal plants (*S. singueana*, *M. azedarach*, *Moringa oleifera* and *L. discolor*) are relatively weaker than TCEA [40]. More importantly, the most potent isolate obtained from cocoa pod husk using ethyl acetate extract only yielded an EC_50_ = 42.7 μg/mL [10]. The superiority of TCEA against *T. cacao* bean extracts were shown in reported studies using n-hexane (EC_50_ = 31.8 μg/mL) [42] and water-methanol—1:1 ratio (EC_50_ = 16.4–53.3 μg/mL) [43]. Furthermore, TCEA is proven more active than the previously reported cocoa pod husk extracted using either ethanol or methanol:acetone [9]. Nevertheless, the same study also revealed higher antioxidant activities of cocoa bean shells and cocoa mucilage than TCEA [9]. These variations of antioxidant activities are ascribed to different phytoconstituents contained in the sample, which are dependent on the solvent, extraction technique and part of the plant. Therefore, the sample with the highest antioxidant activities (TCEA) was screened for its phytoconstituents in GC-MS technique. 

### 3.8. Phytoconstituents 

Dominant bioactive compounds contained in the TCEA, as obtained from the GC-MS analysis, were listed in Table 5. The top five most dominant phytocompounds identified in an increasing order are gamma-sitosterol > stigmasterol > campesterol > methyl octadec-17-ynoate > bis(2-ethylhexyl) phthalate. Gamma-sitosterol, an epimer of beta-sitosterol, is a steroid dominantly contained in medicinal plants of Lagerstroemia spp [44]. In *T. cacao*, beta-sitosterol has been reported to be found in its seed [45,46]. Moreover, the presence of this phytosterol has been found in cocoa pod husk obtained with microwave-assisted extraction [16]. The compound itself has been closely associated with antioxidants and thus can be utilized as an antihyperglycemic [47,48], antidiabetic [49], antidepressant, and anticonvulsant agent [50]. However, recent findings suggested that gamma-sitosterol possesses high toxicity capable of damaging DNA in human peripheral blood mononuclear cells [44]. This compound might be responsible for the high antioxidant activity of TCEA, along with its higher cytotoxicity in comparison with TCW and TCH.

Other common phytosterols (stigmasterol and campesterol) were also identified in the aqueous extract from *T. cacao* pod husk which was estimated to be as much as 20.46 and 5.61%, respectively. Similar findings had been reported in *T. cacao* seeds and pod husk extracts, obtained using non-polar solvent–n-hexane [11,45]. The finding of these compounds (sitosterol, stigmasterol, and campesterol) along with their respective content percentage is in line with that of a previously published study investigating the genus Theobroma [51]. The identified phytosterols are well-known phytoconstituents for their potent antioxidant activity [52] and other medicinal functions [53,54]. Meanwhile, two other compounds which are fatty acid (methyl octadec-17-ynoate) and ester (bis(2-ethylhexyl) phthalate) compounds, are scarcely reported for their bioactivity. Though bis(2-ethylhexyl) phthalate, a commonly used plasticizer, has been reported toxic to humans [55]. Taken together, phytosterol contents in the TCEA were suspected to play a significant role in the biological activities of TCEA reported in this work.

## 4. Conclusions

The aqueous extract of *T. cacao* pod husk has a wide potential of applications in foods stemming from its antimicrobial, antioxidant, and non-toxic properties. Investigations of antimicrobial and antioxidant activities suggest TCEA contains a large number of active compounds. Further phytochemical screening using GC-MS revealed the three dominant phytosterols (gamma-sitosterol, stigmasterol, and campesterol), attributed to the high activity of the sample in scavenging DPPH free radicals.

## Figures and Tables

**Table 1:** Yield and phytochemical contents of cocoa pod husk extracts.

Sample	Yield (%)	Phytochemical Contents					
		Alkaloids	Flavonoids	Terpenoids	Steroids	Saponins	Phenolics
TCW	33.712	+	+	−	−	+	+
TCH	0.047	−	−	+	+	−	−
TCEA	0.134	+	+	+	−	−	+
TCM	0.878	+	+	−	−	+	+
TCWP	89.390	+	+	−	−	+	+

TCW, TCH, TCEA, TCM, and TCWP refers to water extract, n-hexane soluble, ethyl acetate soluble, methanol soluble, and water partition from cocoa pod husk, respectively. The symbols: + and − indicate the presence and absence of the phytochemical content compounds, respectively.

**Table 2:** Antibacterial and antifungal activities of cocoa pod husk extracts.

Sample	Inhibition Diameter (mm)											
	*Staphylococcus aureus*				*Escherichia coli*				*Candida albicans*			
	1%	5%	10%	20%	1%	5%	10%	20%	1%	5%	10%	20%
TCW	−	−	−	−	−	−	−	−	−	−	−	−
TCH	−	−	6.10 ± 0.04 ^a^	6.55 ± 0.04 ^a^	−	−	−	−	−	6.70 ± 0.10	7.73 ± 0.08	9.68 ± 0.02 ^a^
TCEA	−	−	6.14 ± 0.04 ^a^	6.62 ± 0.10 ^a^	−	−	−	6.52 ± 0.02	−	−	−	11.72 ± 0.36 ^b^
TCM	−	−	−	6.38 ± 0.08 ^b^	−	−	−	−	−	−	−	−
Control *	19.41 ± 1				14.61 ± 1.1				13.12 ± 1.08 **			

^a,b^ In one column, values with the same alphabet are not significantly different at *p* < 0.05 using *t*-test.Extract concentration was expressed in % *w*/*v*. * Otherwise stated, the control was gentamicin. ** Ketoconazole. The symbol − indicate the absence of the inhibition.

**Table 3:** Cytotoxicity of cocoa pod husk extracts.

Sample	Mortality (%)					Linear Equation	LC_50_ (μg/mL)
	1 μg/mL	10 μg/mL	100 μg/mL	500 μg/mL	1000 μg/mL		
TCW	33	37	43	43	47	y = 0.116x + 4.567	5495.4
TCH	3	10	20	23	27	y =0.458x + 3.038	19,054.6
TCEA	23	27	33	60	80	y = 0.472x + 4.046	104.7
TCM	20	30	37	43	93	y = 0.562x + 3.948	74.1

**Table 4:** Antioxidant activities of cocoa pod husk extracts.

Sample	Inhibition (%)					EC_50_ (μg/mL)
	6.25 μg/mL	12.5 μg/mL	25 μg/mL	50 μg/mL	100 μg/mL	
TCW	12.74 ± 0.88 ^a^	25.60 ± 0.77 ^a^	28.86 ± 0.23 ^a^	42.16 ± 0.68 ^a^	46.71 ± 0.86 ^a^	97.69 ± 0.46 ^a^
TCH	35.04 ± 0.45 ^b^	35.30 ± 0.39 ^b^	38.44 ± 0.79 ^b^	44.11 ± 0.73 ^a^	46.56 ± 0.70 ^a^	116.70 ± 0.86 ^b^
TCEA	11.84 ± 0.85 ^a^	23.07 ± 0.92 ^c^	33.99 ± 0.68 ^c^	48.30 ± 0.65 ^b^	61.04 ± 0.77 ^b^	9.61 ± 0.64 ^c^
TCM	19.19 ± 0.91 ^c^	23.11 ± 0.86 ^c^	24.56 ± 0.63 ^d^	35.89 ± 0.57 ^c^	46.49 ± 0.95 ^a^	108.33 ± 0.77 ^d^
TCWP	30.04 ± 0.83 ^d^	31.56 ± 0.66 ^d^	37.97 ± 0.99 ^b^	42.49 ± 0.89 ^a^	45.60 ± 0.92 ^a^	115.52 ± 0.78 ^b^

^a,b,c,d^ In one column, values with the same alphabet are not significantly different at *p* < 0.05 using *t*-test. EC_50_ of positive control (ascorbic acid) was 2.804 μg/mL.

**Table 5:** Phytoconstituents of TCEA obtained from GC-MS analysis.

Compound (Formula)	Retention (Min)	Molecular Weight (g/mol)	Area (%)
Dodecanoic acid (C_12_H_24_O_2_)	16.58	200	0.22
Tetradecanoic acid (C_14_H_28_O_2_)	20.27	228	0.20
Heptadecane C_17_H_36_	20.76	240	0.23
1,2-Benzenedicarboxylic acid, bis(2-methylpropyl) ester (C_16_H_22_O_4_)	21.64	278	0.33
n-Hexadecanoic acid (C_16_H_32_O_2_)	22.43	256	1.43
Dibutyl phthalate (C_16_H_22_O_4_)	22.56	278	0.24
Heneicosane (C_21_H_44_)	22.75	296	0.22
1-Nonadecene (C_19_H_38_)	23.46	266	0.12
4a-But-3-enyl-2-t-butyl-tetrahydrocyclopenta[1,3]dioxin-4-one (C_15_H_24_O_3_)	23.62	252	0.14
Octanoic acid, 4,6-dimethyl-, methyl ester, (4S,6S)-(+)- (C_11_H_22_O_2_)	23.76	186	0.14
Octadecane (C_18_H_38_)	24.27	254	0.11
Methyl octadec-17-ynoate (C_19_H_34_O_2_)	24.86	294	2.28
9-t-Butyltricyclo[4.2.1.1(2,5)]decane-9,10-diol (C_14_H_24_O_2_)	24.95	224	0.39
Campesterol (C_28_H_48_O)	25.10	400	5.61
Ethyl stearate, 9,12-diepoxy (C_20_H_36_O_4_)	25.82	340	0.18
Oxiraneoctanoic acid, 3-octyl-, cis- (C_18_H_34_O_3_)	25.94	298	0.18
Stigmasterol (C_29_H_48_O)	26.25	412	20.46
Hexadecanoic acid, 2-hydroxy-1-(hydroxymethyl)ethyl ester (C_19_H_38_O_4_)	26.60	330	0.43
Bis(2-ethylhexyl) phthalate (C_24_H_38_O_4_)	26.96	390	1.82
Cyclononasiloxane, octadecamethyl- (C_18_H_54_O_9_Si_9_)	27.99	666	0.12
gamma-Sitosterol (C_29_H_50_O)	28.42	414	64.74
Stigmastanol C_29_H_52_O	28.80	416	0.27
Decanoic acid, 2-hydroxy-3-[(1-oxooctyl)oxy]propyl ester C_21_H_40_O_5_	29.13	372	0.13

## Data Availability

The data presented in this study are available on request from the corresponding author.

## Sample Availability

Samples of the compounds are not available from the authors.
